# *Burkholderia stabilis* outbreak associated with contaminated commercially-available washing gloves, Switzerland, May 2015 to August 2016

**DOI:** 10.2807/1560-7917.ES.2017.22.49.17-00213

**Published:** 2017-12-07

**Authors:** Rami Sommerstein, Urs Führer, Elia Lo Priore, Carlo Casanova, Dominik M Meinel, Helena MB Seth-Smith, Andreas Kronenberg, on behalf of Anresis, Daniel Koch, Laurence Senn, Andreas F Widmer, Adrian Egli, Jonas Marschall, on behalf of Swissnoso

**Affiliations:** 1 Department of Infectious Diseases, Bern University Hospital, University of Bern, Bern, Switzerland; 2 These authors contributed equally to the manuscript; 3 Infectious Diseases Department, Biel Hospital, Biel, Switzerland; 4 Institute for Infectious Diseases, University of Bern, Bern, Switzerland; 5 Division of Clinical Microbiology, University Hospital Basel, Basel, Switzerland; 6 Applied Microbiology Research Unit, Department of Biomedicine, University of Basel, Basel, Switzerland; 7 Federal Office of Public Health, Bern, Switzerland; 8 Service of Hospital Preventive Medicine, Lausanne University Hospital, Lausanne, Switzerland; 9 Department of Infectious Diseases, University Hospital Basel, Basel, Switzerland; 10 The members of the group are listed at the end of the paper

**Keywords:** Burkholderia cepacia complex, Burkholderia stabilis, outbreak, medical device, whole genome sequencing, nationwide

## Abstract

We describe an outbreak of *Burkholderia stabilis* associated with contaminated washing gloves, a commercially available Class I medical device. Triggered by an increase in *Burkholderia cepacia* complex (BCC) bacteremias and the detection of BCC in unopened packages of washing gloves, an ad hoc national outbreak committee comprising representatives of a public health organisation, a regulatory agency, and an expert association convened and commissioned an outbreak investigation. The investigation included retrospective case finding across Switzerland and whole genome sequencing (WGS) of isolates from cases and gloves. The investigation revealed that BCC were detected in clinical samples of 46 cases aged 17 to 91 years (33% females) from nine institutions between May 2015 and August 2016. Twenty-two isolates from case patients and 16 from washing gloves underwent WGS. All available outbreak isolates clustered within a span of < 19 differing alleles, while 13 unrelated clinical isolates differed by > 1,500 alleles. This BCC outbreak was rapidly identified, communicated, investigated and halted by an ad hoc collaboration of multiple stakeholders. WGS served as useful tool for confirming the source of the outbreak. This outbreak also highlights current regulatory limitations regarding Class I medical devices and the usefulness of a nationally coordinated outbreak response.

## Introduction

Medical devices are defined as any instrument, apparatus, appliance, material or other article to be used on human beings for diagnosis, prevention, monitoring or alleviation of disease. In Europe, medical devices are classified by the potential risk they pose to the health of patients, users or third parties [1,2]. The underlying rationale for this risk-based system is to direct the most rigorous conformity assessment procedures to those products conferring the greatest risk [1,2]. Class I medical devices, e.g. non-sterile products that only come into contact with intact skin [1], represent the lowest risk category and conformity assessment is left to the responsibility of the manufacturer [3]. If a Class I device conforms with the Medical Devices Directive it can then be placed on the market in the entire European Economic Area, Turkey and Switzerland [3,4]. Only a few outbreaks have been traced to Class I medical devices contaminated at the site of their production [5,6].

Bacteria belonging to the *Burkholderia cepacia* complex (BCC) are ubiquitous in the environment and survive with minimal nutritional requirements. These bacteria may cause difficult-to-treat lung infections, for example, in cystic fibrosis patients [7], and outbreaks in the hospital setting [8,9] are often linked to contaminated products used in patient care. Specifically, reports have been published on outbreaks caused by medical devices [10], ultrasound gel [11], environmental contamination [12,13], inhaled products [14-16], parenteral solutions [17-21], disinfectants [22-24], lubricants for urinary catheterization [25], mouthwash [26-30], laxatives [31], products for external use such as moisturizing cream [32,33] and washcloths [6,34].

Here, we present data on an outbreak with *B. stabilis*, a member of the BCC and associated with contaminated washing gloves, a Class I medical device. These washing gloves are commercially available packaged products that are usually acquired by healthcare institutions and intended for whole body washing of bedridden patients.

We also describe the response to the outbreak by an interdisciplinary group of stakeholders, including a public health organisation, a regulatory agency, an expert association and a professional society. Finally, we discuss regulatory and administrative issues limiting efficient and thorough national or even international investigation of an outbreak with a Class I medical device.

### The event

The initial cluster of nosocomial BCC infections was detected in April and May 2016 in a 270-bed regional hospital in the canton of Bern, Switzerland. The index patient was an adult in their 70s with a central venous catheter-associated bloodstream infection due to BCC, as diagnosed on 5 April 2016 when she was in the intensive care unit (ICU). The striking feature of this first case was the occurrence of bacteraemia only two days after admission to the hospital and only 2 days after placement of a central venous catheter. A second case of bloodstream infection with BCC occurred two weeks later in an adult in their 60s with a prolonged stay in the same ICU. These two cases triggered a local outbreak investigation that could not identify the source of the infection. The outbreak continued within the same ICU, with third and fourth bacteraemia cases until 11 May 2016.

On 26 April 2016 a first sampling assessed ultrasound gel and the hospital environment (computer keyboards, resuscitation car, patient monitors, doors of patient rooms) but all remained negative. On 13 May 2016, a second negative sampling included local anaesthesia medication and chlorhexidine alcohol used for preparation of central vein access. Eventually, on 27 May 2016, BCC were discovered in a package of disinfectant-free washing gloves (Sinaqua, Welcare, Italy). These gloves come in different formats (gloves or wipes), and are pre-moisturised. They may also contain chlorhexidine 2%. The gloves implicated here are disinfectant free but contain benzalkonium chloride 0.1% as a preservative agent. On 27 May 2016, the Swiss Agency for Therapeutic Products (Swissmedic) and the Swiss distributor of the washing gloves GD Medical (Freienbach, Switzerland) were informed of these findings. Swissmedic acted within the legal framework and did not impose an immediate sales stop. Shortly thereafter, in June 2016, Bern University Hospital identified a series of patients in whom BCC were detected. Washing glove contamination with BCC, which had been identified as the possible source for infections in the regional hospital, was subsequently also found in Bern on 24 June 2016 following four cases with the same pathogen and exposure to the same gloves from the same manufacturer. On 27 June 2016, Swissmedic ordered an immediate sales stop of the product.

## Methods

### Organisation of the national outbreak investigation

The Swiss Centre for Antibiotic resistance (Anresis) [35] was contacted on 5 July 2016 and commissioned to search their database for non-duplicate *B. cepacia* isolates for the time period from January 2015 to June 2016. Anresis has previously been described in detail [36]. In brief, Anresis prospectively collects routine antibiotic resistance data from 20 clinical microbiology laboratories evenly distributed across Switzerland, representing at least 70% of annual hospitalisation days in Switzerland. Most of the laboratories gather data from multiple hospitals, ranging from primary-care to tertiary-care institutions. Anresis collects data both from inpatients and outpatients. Unpaired t-tests were used to compare monthly numbers of new cases of *B. cepacia* isolates between 2015 and 2016.

On 7 July 2016 as a result of the confirmation that the gloves were involved, a meeting between representatives of Swissmedic, the Federal Office of Public Health (FOPH) and Swissnoso (the Swiss National Center for Infection Prevention) was held. The infection control unit of Bern University Hospital was mandated to conduct the national outbreak investigation. Shortly thereafter on 18 July 2017, the Swiss Society for Infectious Diseases (SSI) sent an alert to all members concerning the contaminated product. Genotyping and sequencing of the isolates was assigned to the Division of Clinical Microbiology at the University Hospital Basel.

### Epidemiologic investigation

Healthcare institutions in Switzerland that ordered any Sinaqua products were identified via a list provided by the Swiss distributor. A case report form and a product report form (see Supplementary Materials [37]) were sent by email on 22 July 2016 to the identified institutions. The email was sent to the following recipients, in descending order, according to their availability in the institutions: hospital epidemiologist or director of infection prevention, chief of the infectious diseases division, or chief of internal medicine. A reminder email containing preliminary results was sent out on 29 August 2016.

#### Case definition, patient and product reporting forms

A case was defined as a patient with a BCC isolate from a clinical specimen obtained since 1 January 2015, and likely clinical contact with Sinaqua Dermal Glove. Only the first non-duplicate isolate was considered. The case report form collected data on patient characteristics, sampling method, storage of clinical BCC isolates for further analyses, product testing for contamination and the corresponding results, details regarding individual patient contact with Sinaqua Dermal Glove, and patient outcomes. The product report form collected data on different Sinaqua products used within the healthcare institutions, the departments where these were used, microbiological tests on the products and the corresponding results, the date the institution became aware of the washing glove contamination, and the delay until complete withdrawal of the washing glove product.

#### International alert

The European Centre for Disease Prevention and Control (ECDC) was informed about the outbreak on 22 September 2016. An urgent inquiry related to this *Burkholderia* outbreak was posted on the Epidemic Intelligence Information System for antimicrobial resistance and healthcare-associated infections (EPIS AMR-HAI), a platform where nominated public health experts across the EU can exchange technical information.

### Microbiological investigation and whole genome sequencing

Evaluation of Sinaqua products (washing glove, wipes, shampoo caps) was performed in several Swiss microbiological laboratories capable of processing environmental samples. If available, data on the product, the lot number, information on whether a package was sealed or had been opened before, and culture results were collected. This data was in addition to what we received via the questionnaire. A descriptive analysis of the results was performed.

DNA extracted from selected strains (22 clinical isolates, 16 glove isolates and 13 unrelated clinical isolates, see Supplementary Materials [37]) underwent library preparation with the Nextera XT Library Preparation Kit (Illumina, San Diego, United States (US)) and sequencing with the MiSeq System (Illumina, San Diego, US), applying v3 2x300 bp chemistry. After read trimming, data was mapped against the reference genome of *B. cepacia* type strain ATCC 25416 (GenBank accession numbers NZ_CP012981–3) within CLC Genomics Workbench 9.5 (Qiagen, Venlo, the Netherlands) to determine genome coverage values [38]. Reads from isolate *E* provided a high coverage (> 100x) and were selected for de novo assembly within CLC Genomics Workbench. Automated annotation of predicted coding sequences was performed with Prokka [39]. The assembled genome was used for species identification, with marker genes extracted and compared with earlier described species using BLASTn [40]. Molecular epidemiological investigations were carried out using core genome multilocus sequence typing (cgMLST) analysis with Ridom SeqSphere+ v3.2.1 (Ridom, Münster, Germany) [41]. Reads were quality trimmed to ≥ Q30 and mapped against the reference genome of *B. stabilis* type strain ATCC27515 BAA-67 (GenBank accession numbers CP016442–4) [42]. A minimum coverage of 10x was needed for mapping. To obtain maximum resolution in resolving the outbreak, all annotated genes were included for allele based typing in Ridom SeqSphere+. Read data for all samples sequenced has been submitted to the European Nucleotide Archive (ENA) under project numbers PRJEB18658 and PRJEB19203.

## Results

### Anresis database search

The results of the Anresis query were available on 5 July 2016. It yielded a non-significant (p = 0.43) increase in non-duplicate *B. cepacia* isolates per month in Switzerland from 5.3/month in 2015 (total n = 64) to 7.2/month in the first six months of 2016 (total n = 39). The 39 isolates identified between 1 January and 5 June 2016 were distributed across eight of all 26 Swiss cantons. Neither age nor gender of the cases was reported by the Anresis query. The number of *B. cepacia* isolates from sterile sites increased non-significantly (p = 0.38) from 0.8/month in 2015 (total n = 10) to 2.2/month in the first six months of 2016 (total n = 13). Of note, there was a peak of six isolates from invasive cases in May 2016.

### Epidemiologic investigation

According to the list from the Swiss distributor of the washing gloves, we identified and contacted 46 healthcare institutions that had purchased any Welcare products. Between 27 July and 21 November 2016 we received 19 responses from these institutions. Ten of 19 reported no cases and/or not using this product any more. From the remaining nine institutions, we received report forms for 50 cases of BCC recovered after January 2015. These nine institutions were scattered across seven of 26 cantons and highly congruent with the cantons identified in the Anresis query, including locations in the eastern, central, northern and western part of the country. They included three of the five Swiss academic hospitals, as well as six midsize to large regional hospitals with 200 to 900 beds.

Of note, all nine institutions with cases employed a physician trained in infectious diseases; in six of these nine institutions, the infectious diseases physician had also undergone training in infection control.

**Figure 1:**
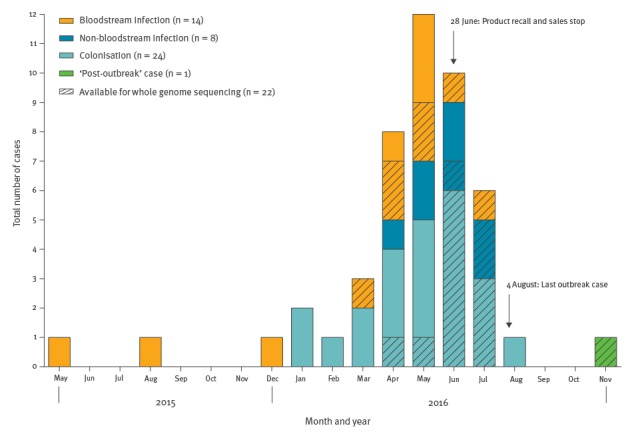
Diagnosis of cases with *Burkholderia cepacia* complex associated with contaminated Sinaqua Dermal Gloves by date of detection and type of infection or colonisation, Switzerland, May 2015–October 2016 (n = 46 outbreak cases and 1 ‘post-outbreak’ case^a^)

^a^ Due to incomplete withdrawal of the washing gloves.

Each of these nine institutions reported between one and nine cases each. After excluding four cases where contact with Sinaqua Dermal Glove was unlikely, a total of 46 cases were further evaluated. Cases were stratified according to the clinical diagnosis related to the recovered isolate. The epidemiologic curve of these cases, including mark-up of cases that underwent WGS, is shown in Figure 1. Patient characteristics, treatment and outcome data, stratified by the clinical diagnosis, and information as to whether the isolate belongs to the outbreak cluster defined by WGS are displayed in Table. The outbreak strain was susceptible to most antimicrobial agents with BCC activity and we opted not to report resistance data.

**Table:** Patient characteristics, treatment and outcome data, and evidence of association with outbreak strain of *Burkholderia stabilis*, Switzerland, May 2015–August 2016 (n = 46)

Characteristic	All Cases (n = 46)	Bloodstream infections (n = 14)	Non-bloodstream infections^a^ (n = 8)	Colonisation (n = 24)
	n or (median)	n or (median)	n or (median)	n or (median)
**Age, years**	(67)	(71)	(69)	(60)
**Female sex**	15	4	4	7
**Unit where contact with Sinaqua occurred**	**46**	**14**	**8**	**24**
ICU	23	7	6	10
Non-ICU medicine	9	3	0	6
Non-ICU surgery	10	4	2	4
Outpatient	4	0	0	4
**Clinical outcome^b^**	**46**	**14**	**8**	**24**
No clinical impact	23	2	0	21
Resolution after antimicrobial treatment	14	5	6	3
Prolonged hospitalisation	7	6	1	0
ICU admission	1	1	0	0
Death	1	0	1	0
**Antimicrobial treatment**	**26**	**12**	**7**	**7**
Broad-spectrum beta-lactam or third/fourth generation cephalosporin	12	4	3	5
Carbapenem	15	6	6	3
Trimethoprim/Sulfamethoxazol	9	8	1	0
Other	2	1	1	0
> 1 therapeutic agent	13	6	4	3
**Case isolates available for whole genome sequencing^c^**	**21**	**7**	**3**	**11**
Isolate belongs to the outbreak cluster	21	7	3	11

ICU: intensive care unit.

**^a^** Non-bloodstream infections included five respiratory tract infections, one wound infection, one urinary tract infection and one case of peritonitis.

**^b^** Only one clinical outcome (worst) per case reported on.

^c^ Does not include the ‘post-outbreak’ case that also underwent whole genome sequencing.

We received completed product report forms from nine institutions, of which eight reported cases. All eight of the responders had been using Sinaqua dermal gloves in their ICUs. Other areas of use included the emergency room, the pre-/post-operative care area, the wound care unit or general wards. Seven institutions had noted the dates when they were first informed of the washing glove contamination and when they withdrew it. The dates of first notice ranged from 27 May to 8 July 2016. The median time until withdrawal was completed was 2 days (range: 1–10). We did not receive any reports on international cases through the ECDC’s EPIS AMR-HAI platform.

### Microbiological investigation and whole genome sequencing

We received information on 31 Sinaqua product samples, from six different institutions and the Swiss distributor that had been evaluated for microbial contamination in five different microbiology laboratories. Of the 22 tests concerning the Sinaqua Dermal Glove, 18 tested positive for BCC. In four of the 18, *Serratia marcescens* was recovered in addition to BCC. The analysis included both packaged and opened products but information to make this distinction was often lacking. Also, one of three Sinaqua Dermal Wipes tested positive for BCC. An additional six reports concerned Sinaqua Shampoo Cap and Sinaqua Paediatrics; these products showed no contamination. The data including the lot numbers is summarised in the Supplementary Materials [37].

**Figure 2:**
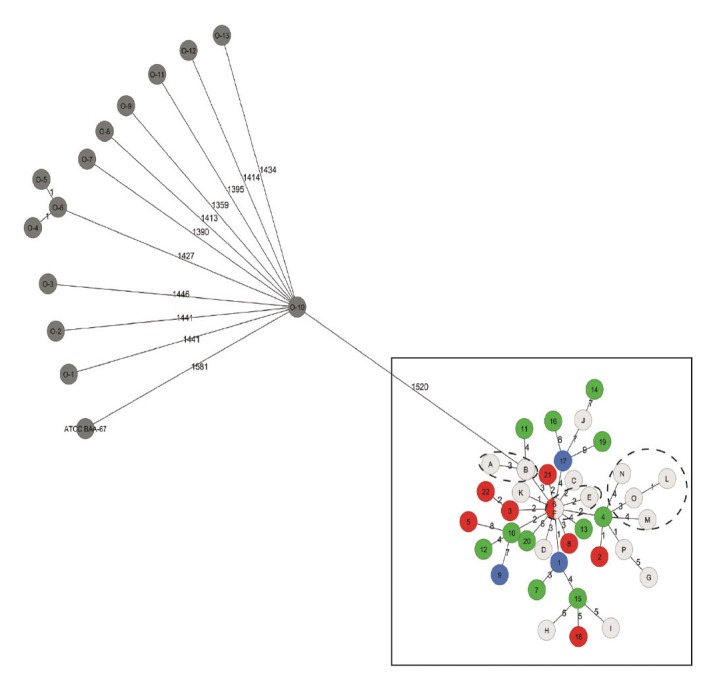
Minimal spanning tree based on cgMLST allelic differences of outbreak and unrelated *Burkholderia* strains from patients and gloves, Switzerland, March 2016–October 2016

There were 22 of 46 isolates from patients meeting the case definition available for WGS (including one ‘post-outbreak’ case because of incomplete withdrawal of the washing gloves), as well as 16 isolates from washing gloves. The genome of outbreak isolate *E* (see Supplementary Materials [37]) was assembled from WGS reads, resulting in a genome draft of 8’300’288 bp across 129 contigs. Marker genes for phylogenetic identification of the species were extracted from the automated assembly and compared against the National Center for Biotechnology Information (NCBI) database [43]. The outbreak strain was identified as *B. stabilis*, best matching *B. stabilis* ATCC BAA-67 according to *recA* (1,065/1,071 identical bp; 99.4%), *rpoB* (4,090/4,107 bp; 99.6%) and *gyrA* (2,543/2,556 bp; 99.5%). Identification using the 16S rRNA gene was less discriminatory, with many species within the BCC sharing over 99% identity, and both *B. stabilis* ATCC BAA-67 and *B.* *pyrrocinia* strain DSM 10685 providing best matches (1,520/1,521 bp; 99.9%). Based on 7,521 predicted coding sequences, cgMLST analysis was performed. Outbreak strains clustered together and differed from each other by a maximum of 18 alleles within the 7,521 coding sequences (Figure 2). The fourteen unrelated *Burkholderia* strains (including the ATC25416 reference strain) sequenced as a part of this project were separated by more than 1,500 alleles from outbreak-associated isolates, as was the *B. stabilis* reference strain ATCC BAA-67. No clustering of strains according to infection or colonisation was observed (Figure 2).

cgMLST: core genome multilocus sequence typing.

Isolates are shown as circles connected to the most related isolates, with numbers of allelic differences depicted along the connecting lines (not to scale). Green circles: isolates from eleven patients that were colonised; red circles: isolates derived from seven patients with bloodstream infections; blue circles: isolates from three patients with non-bloodstream infections; light grey circles: isolates from washing gloves (A-P); dark grey circles: unrelated isolates (O1-O13; ATCC27515 BAA-67). The cluster of outbreak-associated isolates is shown in the box in the lower right. Isolates A and B; E and F; L, M, N, and O, which are grouped by dotted lines, are each from a single, separate package of washing gloves. The core genome multilocus sequence typing (cgMLST) multiple spanning tree was generated in Ridom SeqSphere+ based on 7,521 coding sequences.

## Discussion

We present data on a nationwide outbreak of *B. stabilis* associated with contaminated washing gloves that led to the identification of 46 patients fulfilling the case definition. Among these 46 cases, half were classified as colonisation, while the other half had either a bloodstream or any other infection. The distribution of clinical diagnoses is in line with similar BCC-related outbreaks that were due to contaminated washcloths [6,34]. This association and the widespread availability of these gloves in ICUs could indicate a possible application of the washing glove in central venous catheter care. This, in turn, may have resulted in BCC catheter-related bloodstream infections. Similar to other outbreaks, many items in the patient environment were tested before identifying the contaminated gloves as source of the outbreak (data not shown).

Earlier studies in clinical microbiological diagnostics have shown that the sequence of the 16S rRNA gene is insufficient to define species within the BCC [44,45]. The molecular epidemiological investigation using cgMLST demonstrated limited genomic diversity (up to 18 allelic differences) among the outbreak isolates. This result points to a highly clonal source, which was most likely the manufacturing site. The isolates from patients and washing gloves were interspersed in the minimal spanning tree, indicating that this diversity was already present at the source of the contamination and did not develop during each of the 46 individual manifestations. Interestingly, isolates obtained from a single glove package showed similar variability (2 to 8 allelic differences) as observed between the isolates of different patients. Our observations thus argue for direct colonisation of the patients with the strains contaminating the washing gloves, and reflect the clonal pool of *B. stabilis* within the contaminated glove packages. In line with this finding, we did not identify any clade among the isolates that correlated with colonisation, bloodstream infections or non-bloodstream infection.

This national outbreak highlighted that no single entity in Switzerland was yet entrusted with coordinating such a complex outbreak response. It was only because of the study team noticing detections scattered across multiple cantons that a nationwide outbreak investigation was deemed necessary. Anresis provided an initial search and delineated the trend of increasing *B. cepacia* detections. This was the first time that the Anresis database was used as a supporting tool during an outbreak investigation. The results from the Anresis query demonstrated the importance of a common microbiology laboratory database readily available for access. Our report further suggests that enhancing the Anresis database by adding automated pattern recognition would be an asset to future outbreak investigations.

The ad hoc collaboration between the FOPH, Swissmedic and Swissnoso enabled a swift outbreak investigation. Eventually, a specific infection control unit at Bern University Hospital and a single clinical microbiology laboratory at Basel University Hospital were mandated, although not financially compensated, to conduct this investigation. A framework and reimbursement mechanism would have further streamlined the investigation and as a direct consequence of this outbreak, the FOPH mandated and funded Swissnoso to create and maintain a national outbreak investigation entity.

Swissmedic published a product recall alert on 27 June 2016 and the Swiss distributor sent a letter to customers informing them that all products should be returned to them pending further investigation. The manufacturing company (located in Italy) declared on 25 August 2016 that they had detected the source of contamination in a tube leading to the assembly line, but this was not confirmed by an independent regulatory body and no bacterial isolates from the factory site were available for comparison. Nevertheless, the high genomic relatedness encountered in isolates from both gloves and patients points to the source being most likely the production line. With the recall alert from Swissmedic, the letter from the gloves’ Swiss distributor and a switch to alternative washing gloves, the outbreak was rapidly terminated in August 2016; only one additional case occurred in October 2016 due to incomplete withdrawal of the contaminated product. A follow-up Anresis query on 20 February 2017 documented the decrease in BCC bacteremias to baseline levels after July 2016 and as of November 2017, no additional cases have been reported. We were unable to confirm any cases by WGS that had occurred before March 2016, and the Anresis query did not yield elevated BCC bacteraemia rates before March 2016. We therefore felt that looking back to January 2015 was sufficient. Also, the course of the epicurve makes earlier cases unlikely. Nevertheless, we cannot formally exclude cases that may have occurred before January 2015.

Interactions between Swissmedic and its European counterparts occurred according to the regulatory framework [2,46]. Although Swissmedic announced the product withdrawal on their website and informed the EU’s national competent authorities for medical devices, these public withdrawal alerts may have been poorly visible to the end consumer. For this reason, we directly informed the ECDC on 22 September 2016, which, in response, posted an urgent inquiry on EPIS AMR-HAI. Although no cases were reported to the ECDC to date, we cannot exclude the possibility of additional cases linked to this product having occurred in other countries.

There were several limitations to this study. First, not all institutions identified via the distributor’s list responded to the inquiry. We noted that the nine responding institutions were rather large hospitals with a dedicated infectious diseases service. Second, contact with the washing glove was self-reported and therefore not necessarily accurate. Furthermore, not all isolates were genotyped, especially not those from non-sterile sites, and final proof that they all belong to the outbreak is thus lacking. The extent of the outbreak may also have been underestimated as gloves could have been used in other institutions or in the care of patients where no microbiological sampling was performed. For the outbreak investigation, we did not track infections due to *S. marcescens*, which were sporadically found in the contaminated washing gloves. Finally, because the microbiological evaluation of the washing gloves was sometimes performed on gloves from packages that were already opened or from packages where the status was not available, secondary contamination of the gloves could have been possible. However, the WGS results argue against multiple sources of contamination.

According to the regulatory framework in Switzerland and the EU, only the manufacturing company itself is responsible for taking internal corrective measures for medical Class I devices in response to notifications [2,3,47]. Current legislation does not require a control mechanism of the corrections by an independent regulatory body [2,3,47]. In the authors’ opinion, the processes and legal frameworks for recalling and re-introducing contaminated medical Class I devices should be reviewed in order to further ensure patient safety.

In conclusion, the outbreak was rapidly identified, investigated, communicated and terminated because of an ad hoc, interdisciplinary collaboration between partners at different levels. WGS was a helpful method to describe clonality of the outbreak. For the future, having a national outbreak investigation committee with different stakeholders promises to allow for a rapid outbreak response and independent quality control regulations for contaminated medical devices should be established.

## References

[1] European Commission (EC). Medical Devices: Guidance document. Classification of medical devices. Guidelines relating to the application of the Council Directive 93/42/EEC on medical devices. MEDDEV 2. 4/1 Rev. 9. Luxembourg: EC; June 2010. [Accessed 16 Sep 2016]. Available from: http://ec.europa.eu/consumers/sectors/medical-devices/files/meddev/2_4_1_rev_9_classification_en.pdf

[2] The Swiss Federal Council. Medical Devices Ordinance (MedDO) of 17 October 2001. Bern; the Swiss Federal Council; 1 Jan 2002. [Accessed 12 Dec 2016]. Available from: https://www.admin.ch/opc/en/classified-compilation/19995459/index.html

[3] European Commission. Council Directive 93/42/EEC of 14 June 1993 concerning medical devices. Luxembourg: Publications Office of the European Union. L169. 12 Jul 1993. Available from: http://eur-lex.europa.eu/LexUriServ/LexUriServ.do?uri=CONSLEG:1993L0042:20071011:en:PDF

[4] European Commission. The ‘Blue Guide’ on the implementation of EU products rules 2016. Luxembourg: Publications Office of the European Union. C272. 26 Jul 2016. Available from: http://ec.europa.eu/DocsRoom/documents/12661/attachments/1/translations/en/renditions/native

[5] Iversen BG Eriksen HM Bø G Hagestad K Jacobsen T Engeset E Pseudomonas aeruginosa contamination of mouth swabs during production causing a major outbreak. Ann Clin Microbiol Antimicrob. 2007;6(1):3. 10.1186/1476-0711-6-3 17355630 PMC1831477

[6] Martin M Christiansen B Caspari G Hogardt M von Thomsen AJ Ott E Hospital-wide outbreak of Burkholderia contaminans caused by prefabricated moist washcloths. J Hosp Infect. 2011;77(3):267-70. 10.1016/j.jhin.2010.10.004 21216034

[7] Horsley A Jones AM Lord R Antibiotic treatment for Burkholderia cepacia complex in people with cystic fibrosis experiencing a pulmonary exacerbation. Cochrane Database Syst Rev. 2016; (1):CD009529. 26789750 10.1002/14651858.CD009529.pub3 PMC7100516

[8] Mangram A Jarvis WR Nosocomial Burkholderia cepacia outbreaks and pseudo-outbreaks. Infect Control Hosp Epidemiol. 1996;17(11):718-20. 10.2307/30141542 8934237

[9] Vonberg RP Weitzel-Kage D Behnke M Gastmeier P Worldwide Outbreak Database: the largest collection of nosocomial outbreaks. Infection. 2011;39(1):29-34. 10.1007/s15010-010-0064-6 21153042 PMC7100329

[10] Rutala WA Weber DJ Thomann CA John JF Saviteer SM Sarubbi FA An outbreak of Pseudomonas cepacia bacteremia associated with a contaminated intra-aortic balloon pump. J Thorac Cardiovasc Surg. 1988;96(1):157-61. 3386290

[11] Nannini EC Ponessa A Muratori R Marchiaro P Ballerini V Flynn L Polyclonal outbreak of bacteremia caused by Burkholderia cepacia complex and the presumptive role of ultrasound gel. Braz J Infect Dis. 2015;19(5):543-5. 10.1016/j.bjid.2015.06.009 26322722 PMC9427536

[12] Lucero CA Cohen AL Trevino I Rupp AH Harris M Forkan-Kelly S Outbreak of Burkholderia cepacia complex among ventilated pediatric patients linked to hospital sinks. Am J Infect Control. 2011;39(9):775-8. 10.1016/j.ajic.2010.12.005 21664002

[13] Magalhães M Doherty C Govan JR Vandamme P Polyclonal outbreak of Burkholderia cepacia complex bacteraemia in haemodialysis patients. J Hosp Infect. 2003;54(2):120-3. 10.1016/S0195-6701(03)00118-X 12818585

[14] Balkhy HH Cunningham G Francis C Almuneef MA Stevens G Akkad N A National Guard outbreak of Burkholderia cepacia infection and colonization secondary to intrinsic contamination of albuterol nebulization solution. Am J Infect Control. 2005;33(3):182-8. 10.1016/j.ajic.2005.01.001 15798674

[15] Estivariz CF Bhatti LI Pati R Jensen B Arduino MJ Jernigan D An outbreak of Burkholderia cepacia associated with contamination of albuterol and nasal spray. Chest. 2006;130(5):1346-53. 10.1378/chest.130.5.1346 17099009

[16] Ghazal SS Al-Mudaimeegh K Al Fakihi EM Asery AT Outbreak of Burkholderia cepacia bacteremia in immunocompetent children caused by contaminated nebulized sulbutamol in Saudi Arabia. Am J Infect Control. 2006;34(6):394-8. 10.1016/j.ajic.2006.03.003 16877110

[17] De Smet B Veng C Kruy L Kham C van Griensven J Peeters C Outbreak of Burkholderia cepacia bloodstream infections traced to the use of Ringer lactate solution as multiple-dose vial for catheter flushing, Phnom Penh, Cambodia. Clin Microbiol Infect. 2013;19(9):832-7. 10.1111/1469-0691.12047 23173820

[18] Fernández C Wilhelmi I Andradas E Gaspar C Gomez J Romero J Nosocomial outbreak of Burkholderia pickettii infection due to a manufactured intravenous product used in three hospitals. Clin Infect Dis. 1996;22(6):1092-5. 10.1093/clinids/22.6.1092 8783718

[19] Moehring RW Lewis SS Isaacs PJ Schell WA Thomann WR Althaus MM Outbreak of bacteremia due to Burkholderia contaminans linked to intravenous fentanyl from an institutional compounding pharmacy. JAMA Intern Med. 2014;174(4):606-12. 10.1001/jamainternmed.2013.13768 24493147

[20] Souza Dias MB Cavassin LG Stempliuk V Xavier LS Lobo RD Sampaio JL Multi-institutional outbreak of Burkholderia cepacia complex associated with contaminated mannitol solution prepared in compounding pharmacy. Am J Infect Control. 2013;41(11):1038-42. 10.1016/j.ajic.2013.01.033 23663863

[21] Zorrilla-Vaca A Arevalo JJ Escandón-Vargas K Soltanifar D Mirski MA Infectious Disease Risk Associated with Contaminated Propofol Anesthesia, 1989-2014(1). Emerg Infect Dis. 2016;22(6):981-92. 10.3201/eid2206.150376 27192163 PMC4880094

[22] Ko S An HS Bang JH Park SW An outbreak of Burkholderia cepacia complex pseudobacteremia associated with intrinsically contaminated commercial 0.5% chlorhexidine solution. Am J Infect Control. 2015;43(3):266-8. 10.1016/j.ajic.2014.11.010 25557770

[23] Lee S Han SW Kim G Song DY Lee JC Kwon KT An outbreak of Burkholderia cenocepacia associated with contaminated chlorhexidine solutions prepared in the hospital. Am J Infect Control. 2013;41(9):e93-6. 10.1016/j.ajic.2013.01.024 23608047

[24] Romero-Gómez MP Quiles-Melero MI Peña García P Gutiérrez Altes A García de Miguel MA Jiménez C Outbreak of Burkholderia cepacia bacteremia caused by contaminated chlorhexidine in a hemodialysis unit. Infect Control Hosp Epidemiol. 2008;29(4):377-8. 10.1086/529032 18462153

[25] Ángeles-Garay U Zacate-Palacios Y López-Herrera JR Hernández-Sánchez EA Silva-Sánchez J Ascencio-Montiel IdeJ [Hospital outbreak of urinary tract infections by lubricant gel contaminated with Burkholderia cepacia]. Rev Med Inst Mex Seguro Soc. 2012;50(6):615-22. 23331747

[26] Centers for Disease Control and Prevention (CDC) Nosocomial Burkholderia cepacia infection and colonization associated with intrinsically contaminated mouthwash--Arizona, 1998. MMWR Morb Mortal Wkly Rep. 1998;47(43):926-8. 9822365

[27] Matrician L Ange G Burns S Fanning WL Kioski C Cage GD Outbreak of nosocomial Burkholderia cepacia infection and colonization associated with intrinsically contaminated mouthwash. Infect Control Hosp Epidemiol. 2000;21(11):739-41. 10.1086/501719 11089663

[28] Molina-Cabrillana J Bolaños-Rivero M Alvarez-León EE Martín Sánchez AM Sánchez-Palacios M Alvarez D Intrinsically contaminated alcohol-free mouthwash implicated in a nosocomial outbreak of Burkholderia cepacia colonization and infection. Infect Control Hosp Epidemiol. 2006;27(11):1281-2. 10.1086/508845 17080395

[29] Kutty PK Moody B Gullion JS Zervos M Ajluni M Washburn R Multistate outbreak of Burkholderia cenocepacia colonization and infection associated with the use of intrinsically contaminated alcohol-free mouthwash. Chest. 2007;132(6):1825-31. 10.1378/chest.07-1545 17925414

[30] Martin M Winterfeld I Kramme E Ewert I Sedemund-Adib B Mattner F Ausbruch mit Burkholderia-cepacia-Komplex durch kontaminierte Mundspüllösung. [Outbreak of Burkholderia cepacia complex caused by contaminated alcohol-free mouthwash]. Anaesthesist. 2012;61(1):25-9. German 10.1007/s00101-011-1954-4 22273822

[31] Marquez L Jones KN Whaley EM Koy TH Revell PA Taylor RS An Outbreak of Burkholderia cepacia Complex Infections Associated with Contaminated Liquid Docusate. Infect Control Hosp Epidemiol. 2017;38(5):567-73. 10.1017/ice.2017.11 28166854

[32] Alvarez-Lerma F Maull E Terradas R Segura C Planells I Coll P Moisturizing body milk as a reservoir of Burkholderia cepacia: outbreak of nosocomial infection in a multidisciplinary intensive care unit. Crit Care. 2008;12(1):R10. 10.1186/cc6778 18237375 PMC2374635

[33] Wiener-Well Y Segonds C Mazuz B Kopuit P Assous MV Successful outbreak investigation of Burkholderia cepacia complex bacteremia in intensive care patients. Am J Infect Control. 2014;42(5):580-1. 10.1016/j.ajic.2013.12.015 24655899

[34] Lo Cascio G Bonora MG Zorzi A Mortani E Tessitore N Loschiavo C A napkin-associated outbreak of Burkholderia cenocepacia bacteraemia in haemodialysis patients. J Hosp Infect. 2006;64(1):56-62. 10.1016/j.jhin.2006.04.010 16859809

[35] Swiss Centre for Antibiotic resistance. Bern, Switzerland. [Accessed 12 Dec 2016]. Available from: http://anresis.ch/

[36] Kronenberg A Hilty M Endimiani A Muhlemann K Temporal trends of extended-spectrum cephalosporin-resistant Escherichia coli and Klebsiella pneumoniae isolates in in- and outpatients in Switzerland, 2004 to 2011. Euro Surveill. 2013;18(21):20484. 23725981

[37] Universitätsklinik für Infektiologie. Supplementary Material. Bern: Universitätsklinik für Infektiologie. 3 Jul 2017. Available from: http://www.infektiologie.insel.ch/index.php?id=26760

[38] Daligault HE Davenport KW Minogue TD Bishop-Lilly KA Broomall SM Bruce DC Whole-genome assemblies of 56 burkholderia species. Genome Announc. 2014;2(6):e01106-14. 10.1128/genomeA.01106-14 25414490 PMC4239345

[39] Seemann T Prokka: rapid prokaryotic genome annotation. Bioinformatics. 2014;30(14):2068-9. 10.1093/bioinformatics/btu153 24642063

[40] Altschul SF Gish W Miller W Myers EW Lipman DJ Basic local alignment search tool. J Mol Biol. 1990;215(3):403-10. 10.1016/S0022-2836(05)80360-2 2231712

[41] Jünemann S Sedlazeck FJ Prior K Albersmeier A John U Kalinowski J Updating benchtop sequencing performance comparison. Nat Biotechnol. 2013;31(4):294-6. 10.1038/nbt.2522 23563421

[42] Bugrysheva JV Cherney B Sue D Conley AB Rowe LA Knipe KM Complete Genome Sequences for Three Chromosomes of the Burkholderia stabilis Type Strain (ATCC BAA-67). Genome Announc. 2016;4(6):e01294-16. 10.1128/genomeA.01294-16 27856590 PMC5114382

[43] NCBI Resource Coordinators Database Resources of the National Center for Biotechnology Information. Nucleic Acids Res. 2017;45(D1):D12-7. 10.1093/nar/gkw1071 27899561 PMC5210554

[44] Depoorter E Bull MJ Peeters C Coenye T Vandamme P Mahenthiralingam E Burkholderia: an update on taxonomy and biotechnological potential as antibiotic producers. Appl Microbiol Biotechnol. 2016;100(12):5215-29. 10.1007/s00253-016-7520-x 27115756

[45] Vandamme P Peeters C Time to revisit polyphasic taxonomy. Antonie van Leeuwenhoek. 2014;106(1):57-65. 10.1007/s10482-014-0148-x 24633913

[46] The Swiss Federal Council. Federal Act on Medicinal Products and Medical Devices (Therapeutic Products Act, TPA) of 15 December 2000. Bern: the Swiss Federal Council; 1 Jan 2002. [Accessed 12 Dec 2016]. Available from: https://www.admin.ch/opc/en/classified-compilation/20002716/index.html

[47] European Commission (EC). Medical Devices: Guidance document. Guidelines on a medical devices vigilance system. MEDDEV 2.12-1 rev 8. Luxembourg: EC; January 2013. [Accessed 15 Jun 2017]. Available from: http://ec.europa.eu/DocsRoom/documents/15506/attachments/1/translations

